# Identifying Tissue- and Cohort-Specific RNA Regulatory Modules in Cancer Cells Using Multitask Learning

**DOI:** 10.3390/cancers14194939

**Published:** 2022-10-09

**Authors:** Milad Mokhtaridoost, Philipp G. Maass, Mehmet Gönen

**Affiliations:** 1 Genetics & Genome Biology Program, The Hospital for Sick Children, Toronto, ON M5G 1X8, Canada; 2 Graduate School of Sciences and Engineering, Koç University, İstanbul 34450, Turkey; 3 Department of Molecular Genetics, University of Toronto, Toronto, ON M5S 1A8, Canada; 4 Department of Industrial Engineering, College of Engineering, Koç University, İstanbul 34450, Turkey; 5 School of Medicine, Koç University, İstanbul 34450, Turkey

**Keywords:** cancer, machine learning, miRNAs, mRNAs, multitask learning, RNA regulation

## Abstract

**Simple Summary:**

Understanding the underlying biological mechanisms of primary tumors is crucial for predicting how tumors respond to therapies and exploring accurate treatment strategies. miRNA–mRNA interactions have a major effect on many biological processes that are important in the formation and progression of cancer. In this study, we introduced a computational pipeline to extract tissue- and cohort-specific miRNA–mRNA regulatory modules of multiple cancer types from the same origin using miRNA and mRNA expression profiles of primary tumors. Our model identified regulatory modules of underlying cancer types (i.e., cohort-specific) and shared regulatory modules between cohorts (i.e., tissue-specific).

**Abstract:**

MicroRNA (miRNA) alterations significantly impact the formation and progression of human cancers. miRNAs interact with messenger RNAs (mRNAs) to facilitate degradation or translational repression. Thus, identifying miRNA–mRNA regulatory modules in cohorts of primary tumor tissues are fundamental for understanding the biology of tumor heterogeneity and precise diagnosis and treatment. We established a multitask learning sparse regularized factor regression (MSRFR) method to determine key tissue- and cohort-specific miRNA–mRNA regulatory modules from expression profiles of tumors. MSRFR simultaneously models the sparse relationship between miRNAs and mRNAs and extracts tissue- and cohort-specific miRNA–mRNA regulatory modules separately. We tested the model’s ability to determine cohort-specific regulatory modules of multiple cancer cohorts from the same tissue and their underlying tissue-specific regulatory modules by extracting similarities between cancer cohorts (i.e., blood, kidney, and lung). We also detected tissue-specific and cohort-specific signatures in the corresponding regulatory modules by comparing our findings from various other tissues. We show that MSRFR effectively determines cancer-related miRNAs in cohort-specific regulatory modules, distinguishes tissue- and cohort-specific regulatory modules from each other, and extracts tissue-specific information from different cohorts of disease-related tissue. Our findings indicate that the MSRFR model can support current efforts in precision medicine to define tumor-specific miRNA–mRNA signatures.

## 1. Introduction

Cancer is one of the most leading causes of death globally. Despite the remarkable improvements in cancer therapies, cancer patients remain undiagnosed or mistakenly diagnosed in many cases. This mainly happens when cancer therapy cannot match a specific disease due to insufficient knowledge of molecular mechanisms [1]. There is a consensus among cancer biologists that distinct cancers have various molecular subgroups with unique biological characteristics, which is believed as one of the main reasons for drug resistance and less effectiveness treatments [2,3]. Hence, understanding the molecular mechanism of primary tumor cells and tissues is fundamental to infer the biology of human tumors and predict how the tumors respond to therapies [4].

MicroRNAs (miRNAs) are important non-protein-coding RNA regulators of gene expression by directly or indirectly targeting messenger RNAs (mRNAs), and they are also known to be involved in biological processes that impact the formation, progression and treatment of various cancer types [5]. However, the functional roles of miRNAs and their combinatorial effects as regulatory molecules in cellular processes remain elusive [6,7]. Thus, extracting information of miRNA and mRNA relationships from primary tumors informs about the molecular pathogenesis in the underlying tissue and can provide a deeper understanding of the biological mechanisms of miRNAs in cancer. This helps to provide new strategies for further development and application in clinical settings in terms of early diagnosis and better treatment [8].

The evidence presented thus far, besides similarities in the molecular mechanism of different cancers from the same tissue and the highly correlated nature of genomic data [9], clearly demonstrates the need for establishing accurate computational methods to interpret the regulation of mRNAs by miRNAs in similar tumor tissues.

### 1.1. Previous Studies

Identifying interactions between miRNAs and mRNAs has been improved in recent years due to several proposed computational techniques [10,11,12]. Especially, probabilistic methods [13,14,15] led to reported miRNA–mRNA interactions in cancer. Recently, casual links between miRNAs and mRNAs have been reported [16,17]. However, these studies ignore the effectual common assumption of mRNA regulation by other mRNAs [18]. Hence, we formulated a regulatory module as a small subset of mRNAs correlated with each other, regulated directly or indirectly by a small subset of correlated miRNAs.

Previously, we established a single-task algorithm to identify miRNA–mRNA regulatory modules in cancer [19]. Here, we apply multitask learning [20,21], to improve the model’s predictive performance and to extract biological relevant modules. Multitask learning improves the detection power to identify biomarkers for small sample sizes (i.e., various cancer cohorts) by inferring information from abundant data. Multitask learning has been applied successfully to explore the commonalities between cancer-related tasks and corresponding treatment. Examples of such studies include cancer drug susceptibility prediction [22], cancer survival analysis [23], cancer staging [24], diagnosis-specific genotype–phenotype identification [25], embedding multi-omics data and predicting phenotype profile [26], and identification of cancer drug response biomarkers [27].

In this project, we established a multitask learning sparse regularized factor regression (MSRFR) model to increase the power and consistency of biomarker identification by targeting sample size disparity in different cancer cohorts of the underlying tissue.

### 1.2. Our Contributions

MSRFR efficiently extracts tissue- and cohort-specific miRNA–mRNA regulatory modules of multiple cancer types from a similar origin (i.e., same tissue) using miRNA and mRNA expression profiles of primary tumors. MSRFR was able to simultaneously estimate the effective number of modules for each cancer type (cohort-specific) and for shared modules (i.e., tissue-specific overlaps between cohorts of the same origin), and extract regulatory modules by imposing a low-rank structure and by grouping correlated mechanisms. We applied our algorithm on three sets of cancer cohorts of the same tissue (i.e., blood, kidney, and lung).

The predictive performance of MSRFR and the percentage of regulatory modules with significant survival analysis identified by MSRFR was significantly higher than the single-task algorithm [19], which indicates the higher ability of the proposed model in extracting regulatory modules with biological importance. Moreover, the significance of tissue-specific regulatory modules in survival analysis suggests that our algorithm was able to identify miRNA–mRNA regulatory modules with biological functions in both, the underlying cohorts and the associated tissue. Enrichment analysis and literature validation of identified regulatory modules by MSRFR showed disease-associated and tissue-specific miRNA–mRNA signatures. MSRFR can also be customized to be applied on more than two cohorts from the same origin.

## 2. Materials and Methods

### 2.1. Datasets

In this work, we developed a predictive model that incorporates expression profiles and clinical phenotypes of multiple cancer cohorts into a unified learning framework to identify tissue- and cohort-specific miRNA–mRNA regulatory modules. We used Lymphoid neoplasm diffuse large B-cell lymphoma (DLBC), Acute myeloid leukemia (LAML), Kidney renal clear cell carcinoma (KIRC), Kidney renal papillary cell carcinoma (KIRP), Lung adenocarcinoma (LUAD), and Lung squamous cell carcinoma (LUSC) data sets which are publicly available by the Cancer Genome Atlas (TCGA, https://portal.gdc.cancer.gov, accessed on 10 August 2022). TCGA provided genomic characterizations (including miRNA and mRNA expression profiles) and clinical information of cancer patients. Our computational analyses did not include metastatic tumors since their underlying biology is generally different to primary tumors.

For each cohort, we extracted “BCGSC miRNA Profiling” files for miRNA expression profiles of all primary tumors, which are preprocessed using the unified miRNASeq pipeline of TCGA. We also extracted “HTSeq-FPKM” files for mRNA expression profiles of all primary tumors, which are preprocessed using the unified RNASeq pipeline of TCGA. Since not all patients had both miRNA and mRNA expression profiles, we eliminated samples with only one available expression profile and used the primary tumors only with matched miRNA and mRNA expression profiles. We filtered miRNAs and mRNAs by discarding those that were expressed in less than 50% of the tumors from the analysis. Moreover, we considered matched miRNAs and mRNAs of both cohorts incorporated in each analysis. Hence, the number of miRNAs and mRNAs included in each cancer type reduced to around 500 miRNAs and 17,000 mRNAs on average.

TCGA also provided “Clinical Supplement” files of all patients. To evaluate the biological relevance of the identified miRNA–mRNA regulatory modules, we performed survival analysis in our experiments using extracted survival characteristics of patients (i.e., days to last follow-up for alive patients and days to death for deceased patients).

### 2.2. Problem Definition

We developed a machine learning approach that utilizes expression levels of miRNAs and mRNAs to identify highly correlated miRNA–mRNA regulatory modules in primary tumors to determine similarities between multiple cancer cohorts of the same origin and to uncover tissue-specific signatures. In this study, we are given *K* cohorts indexed by *k*. For each cohort, we are given a training set $D_{k}={\left\{\left(x_{ik},y_{ik}\right)\right\}}_{i=1}^{N_{k}}$, which contains miRNA and mRNA levels of $N_{k}$ tumors, where $x_{ik}\in R^{D}$ and $y_{ik}\in R^{T}$ denote miRNA and mRNA expression profiles of tumor *i* for cancer *k*, respectively. All symbols used in our model (in Section 2.3) are described in Table 1.

### 2.3. Method

It is believed that the relationship between miRNAs and mRNAs is sparse [28]. Hence, we formulated the proposed problem as a linear factor multivariate regression model with a low-rank structure on the coefficient matrix to support this assumption. For a single cancer cohort, this formulation can be shown as follows:

(1) $$\begin{array}{cc} Y & =X\overset{W}{\overset{︷}{W_{X}W_{Y}}}+E, \end{array}$$

where $W_{X}\in R^{D\times R}$ projects *D*-dimensional miRNA profiles into a *R*-dimensional space, $W_{Y}\in R^{R\times T}$ performs linear regression in this *R*-dimensional projected space, $E\in R^{D\times T}$ is the matrix of error terms, and $W\in R^{D\times T}$ is the coefficient matrix of regression model. In addition to $W_{X}$ and $W_{Y}$ matrices, the dimensionality of the projected space (i.e., *R*) must to be estimated in the learning process. With this low-rank assumption, instead of learning W matrix, we attempted to learn $W_{X}$ and $W_{Y}$ matrices. By doing so, we were able to reduce the number of parameters that needed to be learned. Furthermore, capability of converting $W_{X}$ and $W_{Y}$ matrices into a summation of rank-one matrices is the other major incentive of this low-rank assumption. Each of these rank-one matrices will be considered as a distinct miRNA–mRNA regulatory module.

We explored a multitask variant of multivariate regression between miRNA and mRNA expression profiles of multiple cancer cohorts under the assumption that the columns of X and Y are centered (i.e., columns with zero mean) and normalized (i.e., columns with unit standard deviation). The error terms in E assumed to be independent and identically distributed Gaussian random variables with zero mean and $\sigma^{2}$ variance.

Considering that the total number of responses (mRNAs) and predictors (miRNAs) are much larger than *N*, but the number of important factors is typically smaller than *N*, it is a credible assumption that the relationship between predictors and responses is sparse. To fit the model on such data, regularized or penalized methods are needed to perform dimensionality reduction and feature extraction. Moreover, we intend to extract similarities of regulatory modules in different tasks (cancer cohorts) which will be interpreted as tissue-specific miRNA–mRNA regulatory modules. To capture this similarity, a multitask learning formulation needs to be applied. Consequently, we proposed MSRFR to find tissue- and cohort-specific miRNA–mRNA regulatory modules of multiple cancer cohorts from their miRNA and mRNA expression profiles and estimate the effective number of regulatory modules as follows:

(2) $$\begin{array}{cc} \mathrm{minimize}\;\; & \frac{1}{2}\sum_{k=1}^{K}{∥Y_{k}-X_{k}\left[W_{XS}W_{Xk}\right]{\left[W_{YS}^{⊤}W_{Yk}^{⊤}\right]}^{⊤}∥}_{F}^{2}\\ \; & +\lambda_{1}(∥W_{XS}{∥}_{1,1}+\sum_{k=1}^{K}∥W_{Xk}{∥}_{1,1})+\lambda_{2}(∥W_{XS}{∥}_{F}^{2}+\sum_{k=1}^{K}∥W_{Xk}{∥}_{F}^{2})\\ \; & +\lambda_{3}(∥W_{YS}{∥}_{1,1}+\sum_{k=1}^{K}∥W_{Yk}{∥}_{1,1})+\lambda_{4}(∥W_{YS}{∥}_{F}^{2}+\sum_{k=1}^{K}∥W_{Yk}{∥}_{F}^{2})\\ \mathrm{with}\;\mathrm{respect}\;\mathrm{to}\;\; & W_{XS}\in R^{D\times R_{s}},\;\;{\left\{W_{Xk}\right\}}_{k=1}^{K}\in R^{D\times R_{k}},\;\;W_{YS}\in R^{R_{s}\times T},\\ \; & {\left\{W_{Yk}\right\}}_{k=1}^{K}\in R^{R_{k}\times T} \end{array}$$

where $\left\{W_{XS},W_{YS}\right\}$ and ${\left\{W_{Xk},W_{Yk}\right\}}_{k=1}^{K}$ are the model parameters that infer the tissue-specific and *K* cohort-specific regulatory modules, respectively. $Y_{k}$ and $X_{k}$ denotes mRNA and miRNA expression profiles of cohort *k*, respectively, $\left\{\lambda_{1},\lambda_{2}\right\}\in R_{+}$ are the user-defined regularization parameters of the elastic net penalty on $W_{XS}$ and ${\left\{W_{Xk}\right\}}_{k=1}^{K}$ matrices, likewise, $\left\{\lambda_{3},\lambda_{4}\right\}\in R_{+}$ are regularization parameters on $W_{YS}$ and ${\left\{W_{Yk}\right\}}_{k=1}^{K}$ matrices, to restrict the search space by enforcing the sparsity structure on the variables based on the input data size. $R_{s}$ and ${\left\{R_{k}\right\}}_{k=1}^{K}$ are the number of shared regulatory modules (i.e., tissue-specific) and cohort-specific regulatory modules of cancer *k*, respectively. Note that number of regulatory modules are chosen a priori before optimization such that $1\leq R_{s},$ ${\left\{R_{k}\right\}}_{k=1}^{K}\leq R_{U}\leq \min\left(D,T\right)$, where parameter $R_{U}$ is the problem-specific upper bound for the dimensionality of the projected space.

**Figure 1:**
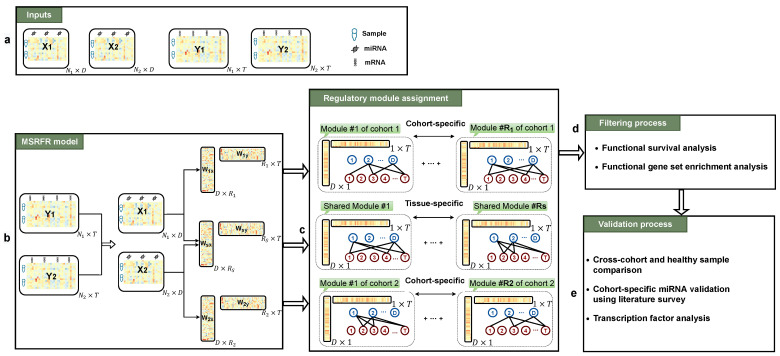
Overview of the developed framework for identifying key tissue-specific and cohort-specific regulatory modules of two cancer types from the same origin. (**a**) Input data that includes miRNA and mRNA expression profiles of two cancer cohorts. (**b**) Imposing low-dimensional structure and multitask learning formulation simultaneously to identify cohort-specific regulatory modules of two cancer types and shared regulatory modules between them (i.e., tissue-specific), as well as effective estimation of the number of each regulatory module (i.e., $R_{1},R_{2}$, and $R_{s}$) using MSRFR model. (**c**) Writing the low-rank matrices as the summation of $R_{1},R_{2}$, and $R_{s}$ rank-one matrices such that each of them corresponds to one miRNA–mRNA regulatory module of first cancer cohort, second cancer cohort, and tissue-specific regulatory modules. (**d**) Filtering identified regulatory modules to test for key modules with biological relevance and functional importance by applying functional survival and gene set enrichment analyses. (**e**) Validating the result of the experiment, first by comparing the result of the study against cross-cohort and healthy sample result to examine the uniqueness of identified modules, then by literature validation of identified miRNAs in cohort-specific regulatory modules to see whether they are related to the underlying disease, and finally by assessing the transcription factor expression in identified modules.

By imposing elastic penalty which linearly combines $\ell_{1}$ and Frobenius norms, as regularization function, we expect two highly correlated features both exist or both absent in a factor. In other words, elastic net penalty empowered our model to group correlated miRNAs together and correlated mRNAs together, besides inducing the sparse structure. In addition to accurately extract all cancers’ miRNA–mRNA regulatory modules separately by inferring information from all data sets, proposed multitask learning formulation that uses shared parameters between all tasks ($W_{XS}$ and $W_{YS}$), which enabled our model to extract joint regulatory modules from all cohorts to be interpreted as tissue-specific miRNA–mRNA regulatory modules. The overall view of the developed framework for two cohorts (i.e., K=2) is demonstrated in Figure 1. To solve the regularized model, as well as to find the number of effective latent factors (i.e., number of regulatory modules), an alternating optimization algorithm was proposed.

Selecting a large or small number of latent factors (i.e., $R_{s},{\left\{R_{k}\right\}}_{k=1}^{K}$ values) would lead to overfitting or underfitting, respectively. To avoid this, we applied the mechanism proposed by [29] that guarantees identifying linearly independent modules and learns how many independent latent factors are needed to explain the data. Since problem (2) is non-convex, it is not expected to find the exact solution in a reasonable time. However, with predefined $R_{s}$ and ${\left\{R_{k}\right\}}_{k=1}^{K}$ values, problem (2) becomes convex if either $\left\{W_{XS},{\left\{W_{Xk}\right\}}_{k=1}^{K}\right\}$ or $\left\{W_{YS},{\left\{W_{Yk}\right\}}_{k=1}^{K}\right\}$ is fixed. This attribute enabled us to apply a heuristic algorithm using a gradient descent method.

To solve problem (2) with predefined $R_{s}$ and ${\left\{R_{k}\right\}}_{k=1}^{K}$ values, we performed Algorithm A1 with a random initial values of decision variables. We determined the stopping criterion of the algorithm according to the objective function of the optimization problem (2). We assumed optimization problem (2) terminates, if $|f^{\left(t+1\right)}-f^{\left(t\right)}|/f^{\left(t\right)}<\epsilon$, where $f^{\left(t+1\right)}$ and $f^{\left(t\right)}$ are the objective function values of problem (2) in the last two iterations.

Our algorithm starts by fixing the number of latent factors with an initial upper bound (i.e., $R_{s}={\left\{R_{k}\right\}}_{k=1}^{K}=R_{U}$). Problem (2) is not jointly convex with respect to all variables, but if we fix $\left\{W_{XS},{\left\{W_{Xk}\right\}}_{k=1}^{K}\right\}$, it will be convex with respect to $\left\{W_{YS},{\left\{W_{Yk}\right\}}_{k=1}^{K}\right\}$ or vice versa. After converting optimization problem (2) to a convex problem by fixing one set of variables, the algorithm starts solving it using an alternating optimization strategy. After convergence, the algorithm checks whether all variable matrices are full rank. If there was any matrix that is not full rank, the algorithm reduces the value of related matrices ranks by one and solves the optimization problem (2) again. For instance, after convergence if $\mathrm{rank}(W_{Xi})$ or $\mathrm{rank}\left(W_{Yi}\right)<R_{i},\forall i\in \left\{S,1,2,...,K\right\}$, then the algorithm reduces $R_{i}$ by one and starts from the first step. The algorithm is guaranteed to achieve full rank matrices (i.e., $W_{XS}$, $W_{YS}$, and ${\left\{W_{Xk},W_{Yk}\right\}}_{k=1}^{K}$), at the termination.

To update variables in each iteration, a gradient descent approach was performed, and to accelerate the convergence of gradient descent, we applied Prox-Linear update [30]. To simplify the notation of update steps, *k* index refers to all k∈{1,⋯,K} for the following steps, and we considered the following notation:

$$\begin{array}{cccc} W_{XSk} & =\left[\begin{array}{cc} W_{XS} & W_{Xk} \end{array}\right] & W_{YSk} & ={\left[\begin{array}{cc} W_{YS}^{⊤} & W_{Yk}^{⊤} \end{array}\right]}^{⊤}. \end{array}$$

The prox-Linear update functions defined as follows:

(3a)WXS(t+1)←Sλ1/αtW˜XS(t)−hW˜XS(t),WYS(t+1)/αs(t)(3b)WXk(t+1)←Sλ1/αtW˜Xk(t)−hW˜Xk(t),WYk(t+1)/αk(t)(3c)WYS(t+1)←Sλ3/βtW˜YS(t)−gWXS(t),W˜YS(t)/βs(t)(3d)WYk(t+1)←Sλ3/βtW˜Yk(t)−gWXk(t),W˜Yk(t)/βk(t)

where *S* is the soft-thresholding function, such that $S_{\tau }\left(\nu \right)=\mathrm{sign}\left(\nu \right)\times \max\left(|\nu |-\tau ,0\right)$, and

$$\begin{array}{cc} \; & h\left(W_{XS},W_{YS}\right)=\sum_{k=1}^{K}\left(X_{k}^{⊤}X_{k}W_{XSk}W_{YSk}W_{YS}^{⊤}-X_{k}^{⊤}Y_{k}W_{YS}^{⊤}\right)+2\lambda_{2}W_{XS}\\ \; & h\left(W_{Xk},W_{Yk}\right)=-X_{k}^{⊤}Y_{k}W_{Yk}^{⊤}+X_{k}^{⊤}X_{k}W_{XSk}W_{YSk}W_{Yk}^{⊤}+2\lambda_{2}W_{Xk}\\ \; & g\left(W_{XS},W_{YS}\right)=\sum_{k=1}^{K}\left(W_{XS}^{⊤}X_{k}^{⊤}X_{k}W_{XSk}W_{YSk}-W_{XS}^{⊤}X_{k}^{⊤}Y_{k}\right)+2\lambda_{4}W_{YS}\\ \; & g\left(W_{Xk},W_{Yk}\right)=-W_{Xk}^{⊤}X_{k}^{⊤}Y_{k}+W_{Xk}^{⊤}X_{k}^{⊤}X_{k}W_{XSk}W_{YSk}+2\lambda_{4}W_{Yk} \end{array}$$

are the derivatives of the objective value of problem (2) without the $\ell_{1}$-penalties with respect to variables (detailed equations are available in Appendix A). Moreover, $\alpha_{s}^{\left(t\right)}$ and $\alpha_{k}^{\left(t\right)}$ are the multipliers that have to be greater than the Lipschitz constant (i.e., the smallest non-negative constant value that satisfies the Lipschitz condition) of $h(W_{XS},W_{YS}^{\left(t+1\right)})$ and $h(W_{Xk},W_{Yk}^{\left(t+1\right)})$, respectively, and $\beta_{s}^{\left(t\right)},\beta_{k}^{\left(t\right)}$ are the multipliers that have to be greater than the Lipschitz constant of $g(W_{XS},W_{YS})$ and $g(W_{Xk},W_{Yk})$, respectively. According to the Equations (A1)–(A4) in Appendix B, the multipliers can be set as follows:

$$\begin{array}{cc} \alpha_{s}^{\left(t\right)} & =\sum_{k=1}^{K}∥X_{k}^{⊤}X_{k}W_{Xk}{∥}_{F}{∥W_{YSk}^{\left(t+1\right)}W_{YS}^{⊤(t+1)}∥}_{F}+2\lambda_{2}\\ \alpha_{k}^{\left(t\right)} & =∥X_{k}^{⊤}X_{k}W_{XS}{∥}_{F}{∥W_{YSk}^{\left(t+1\right)}W_{Yk}^{⊤(t+1)}∥}_{F}+2\lambda_{2}\\ \beta_{s}^{\left(t\right)} & =\sum_{k=1}^{K}{∥W_{XS}^{⊤(t)}X_{k}^{⊤}X_{k}{\left[W_{XS}\;W_{Xk}W_{Yk}\right]}^{\left(t\right)}∥}_{F}+2\lambda_{4}\\ \beta_{k}^{\left(t\right)} & =∥W_{Xk}^{⊤(t)}X_{k}^{⊤}X_{k}{\left[W_{XS}W_{YS}\;W_{Xk}\right]}^{\left(t\right)}{∥}_{F}+2\lambda_{4}. \end{array}$$

Our optimization strategy is described in Algorithm A1 with more details The algorithm’s pseudocode is presented in Appendix C).

For the sake of simplicity, we refer to $W_{XS}$ and ${\left\{W_{Xk}\right\}}_{k=1}^{K}$ as $W_{X}$ and refer to $W_{YS}$ and ${\left\{W_{Yk}\right\}}_{k=1}^{K}$ as $W_{Y}$. Similarly, we also refer to $R_{S}$ and ${\left\{R_{k}\right\}}_{k=1}^{K}$ as *R*. After finding $W_{X}$ and $W_{Y}$ matrices using the proposed algorithm, we need to extract key miRNA–mRNA regulatory modules. We first determined weights of each identified regulatory module. Hence, we normalized each row of $W_{X}$ and each column of $W_{Y}$ to unit norm, in order to set a unified scale for all of the regulatory modules. For this purpose, instead of our initial decomposition assumption (i.e., $W\approx W_{X}W_{Y}$), for each tissue- and cohort-specific regulatory modules we obtained the low rank decomposition $W\approx {\tilde{W}}_{X}D_{X}D_{Y}{\tilde{W}}_{Y}$, where $D_{X}$ and $D_{Y}$ are R×R diagonal matrices. The diagonal entries of $D_{X}$ and $D_{Y}$ are used for assigning the importance weight of each underlying regulatory module. To determine weights of regulatory modules, we sorted the rows of $W_{X}$ and the columns of $W_{Y}$ from the largest one to the smallest one. The regulatory module with the highest importance (i.e., the first regulatory module) refers to the one that corresponds to the highest diagonal entry. The first regulatory module is considered in biological relevance analyses (Section 3), since it reflects the most considerable portion of knowledge.

Similar to our previous strategy [12], we detected miRNAs (mRNAs) for each module as follows: A miRNA (mRNA) is considered to be selected if the magnitude of its weight is larger than two over square root of the total number of miRNAs (mRNAs) included.

To pick the key regulatory modules with biological relevance among all identified regulatory modules, we filtered the regulatory modules by performing functional survival and functional gene set enrichment analyses [31].

First, to check if the MSRFR-identified mRNAs are related to the survival rate of all patients, we classified patients into two groups using *k*-means clustering based on the mRNA expression profiles. We then examined survival rates in the clinical parameters by checking whether there is a statistically significant difference between the two groups of patients using the log-rank test. Significant survival difference refers to a module with different mRNA expression levels of patients of the underlying cohort. The main motivation of this process was exploring the functional importance of identified regulatory modules.

In the second step of filtering, regulatory modules that seem to have biological relevance from filtering step one were further evaluated in functional gene set enrichment analysis [31].

A regulatory module is categorized as a key regulatory module if “survival analysis of the corresponding module reports a significant difference between patient groups” and “underlying module is enriched in either tissue-specific or cohort-specific gene sets”.

Following the biological validation strategy, we attempted to investigate the identifying transcription factors (TFs) in miRNA–mRNA signatures. To determine whether or not TF regulation is affected by cancer-specific miRNA–mRNA signatures, we assessed the currently known human TFs [32] in the regulatory modules. We performed 10,000× permutation analysis to estimate the distribution for the number of TFs in a certain number of random genes and compare it with the number of TFs in the selected mRNAs by MSRFR (Section 3.6).

### 2.4. Experimental Setting

To test the applicability of our MSFRF model, we applied it on three pairs of cancer cohorts where each originated from the same tissue (blood, kidney, lung).

Specifically, Lymphoid neoplasm diffuse large B-cell lymphoma (DLBC) and Acute myeloid leukemia (LAML) cohorts, originated from blood, whilst kidney renal clear cell carcinoma (KIRC) and Kidney renal papillary cell carcinoma (KIRP) occur in kidney, and Lung adenocarcinoma (LUAD) and Lung squamous cell carcinoma (LUSC) derive from lung tissue.

To set reasonable values to hyper-parameters of Problem (2), i.e., $\lambda_{1}$, $\lambda_{2}$, $\lambda_{3}$ and $\lambda_{4}$, we considered five values for each parameter based on the size of underlying cohorts in each experiment. In blood experiments, we considered set {60,80,100,125,150} for $\lambda_{1}$ and $\lambda_{3}$ parameters, and set {10,15,20,25,30} for $\lambda_{2}$ and $\lambda_{4}$ parameters. In kidney and lung experiments, we considered set {150,200,250,300,350} for $\lambda_{1}$ and $\lambda_{3}$ parameters, and set {10,15,20,25,30} for $\lambda_{2}$ and $\lambda_{4}$ parameters.

We trained the algorithm with all combinations of predefined parameters using four-fold cross-validation. Then, we calculated the average root mean square error (RMSE) of four folds between predicted and actual mRNA expression levels for all parameter combinations. For each experiment, we picked the set of parameters with the minimum average RMSE. Finally, the parameters of the three experiments are set as follows:

- Blood $\to \left(\lambda_{1}\lambda_{2},\lambda_{3},\lambda_{4}\right)=\left(80,20,125,15\right)$,
- Kidney $\to \left(\lambda_{1}\lambda_{2},\lambda_{3},\lambda_{4}\right)=\left(300,25,300,30\right)$,
- Lung $\to \left(\lambda_{1}\lambda_{2},\lambda_{3},\lambda_{4}\right)=\left(250,15,300,30\right)$.

We considered $R_{U}=10$ as the upper bound on the number of latent factors in all cohorts of experiments. Thus, due to the number of independent factors that can explain each cohort/tissue, MSRFR identifies 10 or less than 10 regulatory modules. We also set the stopping criteria parameter ϵ to $10^{-4}$, and the maximum number of iterations to $10^{3}$. Since miRNA and mRNA expression profiles of primary tumors are count data and can take only non-negative values, we applied $\log_{2}$-transformation before feeding them to the algorithm. We implemented our algorithm in R.

## 3. Results

### 3.1. Predictive Performance Comparison

We tested the performance of MSRFR by calculating the normalized root mean squared error (NRMSE) of our algorithm between observed and predicted values of mRNA expression levels against the single-task algorithm [19] using

$$\begin{array}{c} {\hat{Y}}_{k}=X_{k}\left[W_{XS}\;W_{Xk}\right]{\left[W_{YS}^{⊤}\;W_{Yk}^{⊤}\right]}^{⊤} \end{array}$$

where *k* indexes the cancer cohorts.

**Figure 2:**
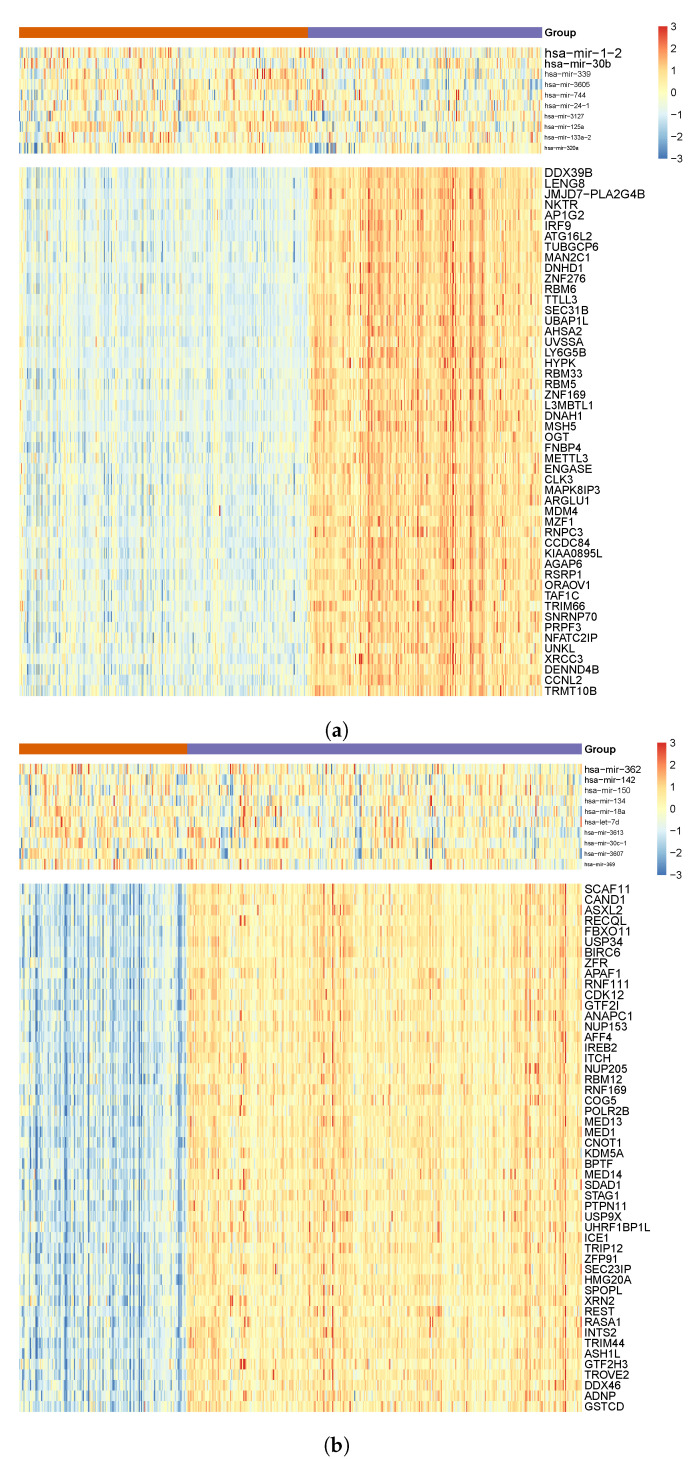
Heat map of top 10 miRNAs and top 50 mRNAs of as (**a**) cohort-specific regulatory module identified in KIRC and (**b**) tissue-specific regulatory module identified for kidney, clustered into two groups of patients using *k*-means clustering on mRNA expression values. Red colors indicate over-expression (i.e., higher than the population mean), and blue colors indicate lower expression (i.e., lower than the population mean). The font sizes of miRNAs and mRNAs are proportional to the magnitudes of their weights inferred by our algorithm.

The predictive performance comparison showed that MSRFR algorithm explained a higher proportion of variance than the single-task algorithm in five out of six cohorts using fewer regulatory modules (Table 2). By applying MSRFR in three experiments to cancer cohorts of blood, kidney, and lung, we identified 75 regulatory modules in total (12 cohort-specific and 10 tissue-specific modules for blood, 14 cohort-specific and 10 tissue-specific modules for kidney, and 19 cohort-specific and 10 tissue-specific modules for lung). Figure 2a,b show examples for selected top 10 miRNAs and top 50 mRNAs identified in the first regulatory modules of KIRC cohort and kidney tissue, respectively. The first regulatory module refers to the extraction of the most significant result from each cohort/tissue. Selected miRNAs and mRNAs for all regulatory modules identified by MSRFR are presented in Supplementary Table S1.

### 3.2. Functional Survival Analysis of Identified Regulatory Modules

To assess the findings of our MSRFR algorithm, we compared results between different cohorts and tissues. By including the clinical parameters (i.e., survival of patients), we clustered patients into two groups by applying *k*-means clustering on the expression values of the selected mRNAs. Next, we acquired the expression values of selected miRNAs and mRNAs and used the expression profiles of both cancers in each tissue to cluster the patients and to address their survival rates (Figure 2a,b). Significant differences were determined using the log-rank test.

**Figure 3:**
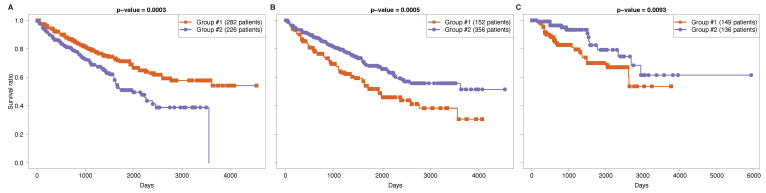
Kaplan-Meier survival curves of two patient groups identified using *k*-means clustering algorithm for (**A**) cohort-specific regulatory module identified in KIRC cohort, and tissue-specific regulatory module identified for kidney using the same subgroup of selected mRNAs in (**B**) KIRC and (**C**) KIRP patients.

For tissue-specific analysis, we selected the overlap of regulatory modules with significant differences in both cancer cohorts of the same tissue. Interestingly, we find that the differences in expression levels of the identified miRNA–mRNA signatures seem to relate to survival differences in the different cancer patient groups (Figure 3). These figures indicated that the identified regulatory module is highly effective in capturing the biological mechanism of both cohorts, and it is associated with kidney tissue.

In 27 of 75 identified regulatory modules in total (36%), we observed a significant survival difference between the two groups (i.e., *p*-value < 0.05 in the log-rank test). To demonstrate the detection power of clinical characterization using the proposed method, we compared the percentage of regulatory modules with significant survival differences in this study against another algorithm [19]. Table 3 shows all identified regulatory modules and those with significant survival differences by MSRFR model and our recently reported single-task algorithm [19]. The percentage of significant survival analysis is higher in MSRFR algorithm, even though, in MSRFR we have two filters for survival analysis of tissue-specific regulatory modules using the expression value and clinical information of both underlying cohorts.

### 3.3. Tissue-Specificity and Disease Association of Key Regulatory Modules

**Figure 4:**
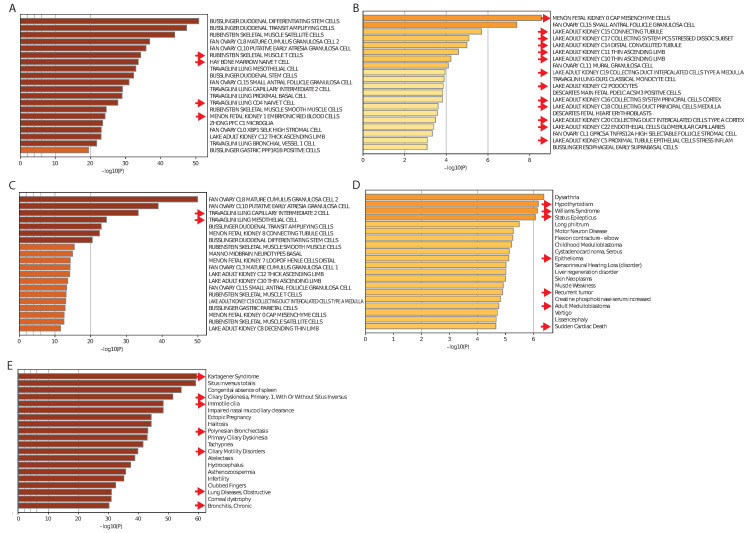
Summary of enrichment analysis in cell types of tissue-specific regulatory modules of (**A**) blood, (**B**) kidney and (**C**) lung. Examples of enrichment analysis of tissue-specific regulatory modules of (**D**) KIRP and (**E**) LUAD in DisGeNET. Red arrows in (**A**–**C**) depict cell-type and tissue-related terms and disease-relevant terms in (**D**,**E**).

We next performed enrichment analysis of selected mRNAs in the first regulatory modules by using cell type signatures [33] and disease association [34] to address if the underlying tissues in tissue-specific regulatory modules and signatures that are cohort-specific can be found. Of note, we observed specific cell type signatures in all tissue-specific modules that related to the underlying tissues (Figure 4A–C).

Regarding disease association of cohort-specific regulatory modules, we found disease associated terms that are relevant to the cohort-specific regulatory modules (Figure 4D,E), thereby validating that MSRFR can specifically determine tissue-specific and cohort-specific miRNA–mRNA signatures.

### 3.4. Comparing miRNA–mRNA Signatures in Cross-Cohort Combinations and with Healthy Samples

**Figure 5:**
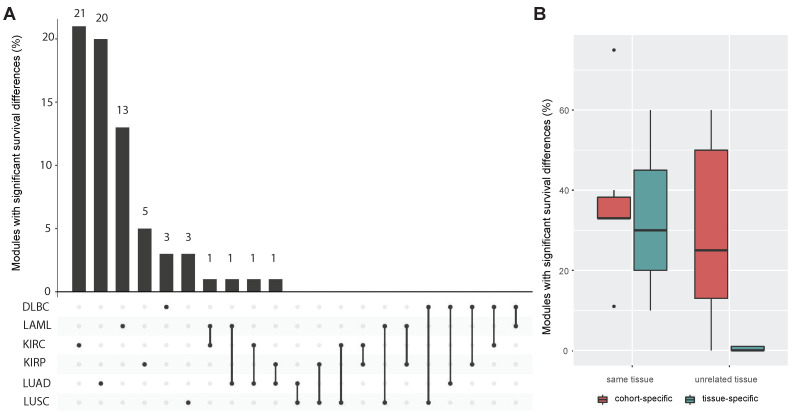
(**A**) Number of cohort-specific and tissue-specific regulatory modules in cross-cohort experiments with significant survival analysis based on clinical information. (**B**) Box plot demonstrating the percentage of tissue- and cohort-specific regulatory modules with significant survival analysis in experiments with cohorts from the same tissue and unrelated tissues.

Next, we examined the uniqueness of identified modules by applying the MSRFR algorithm on a total of 12 combinations of cohorts that did not originate from the same tissue (DLBC–KIRC, DLBC–KIRP, DLBC–LUAD, DLBC–LUSC, LAML–KIRC, LAML–KIRP, LAML–LUAD, LAML–LUSC, KIRC–LUAD, KIRC–LUSC, KIRP–LUAD, and KIRP–LUSC). We found 118 tissue-specific modules and 220 cohort-specific modules in a total of 12 experiments. Of note, only 4/118 (3.39%) of tissue-specific regulatory modules presented significant survival differences, while 64/220 (29.10%) of cohort-specific regulatory modules are significant in survival analysis (Figure 5A). The ratio of significant survival differences in the related cohort experiments was 10/30 (33.33%) and 17/45 (37.78%) for tissue-specific and cohort-specific regulatory modules, respectively (Table 3).

Accordingly, survival analysis based on clinical information in non-relevant cohort pairs was significantly lower than identified regulatory modules with significant survival differences in tissue-specific regulatory modules. However, there is no significant difference in cohort-specific regulatory modules (Figure 5B). These findings indicate that MSRFR identifies tissue-specific regulatory modules with biological relevance using cohorts from the same tissue rather than miRNA–mRNA signatures from non-related tissues.

We conclude that MSRFR increases the detection power of the model by multitask formulation, enhances the capacity of the model in grouping genes that participate in the same processes while extracting fewer modules, effectively determines biologically related miRNA–mRNA regulatory modules by inferring information from other tasks and it distinguishes tissue- and cohort-specific regulatory modules from each other to extract tissue-specific information from different cohorts of disease-related tissue.

**Figure 6:**
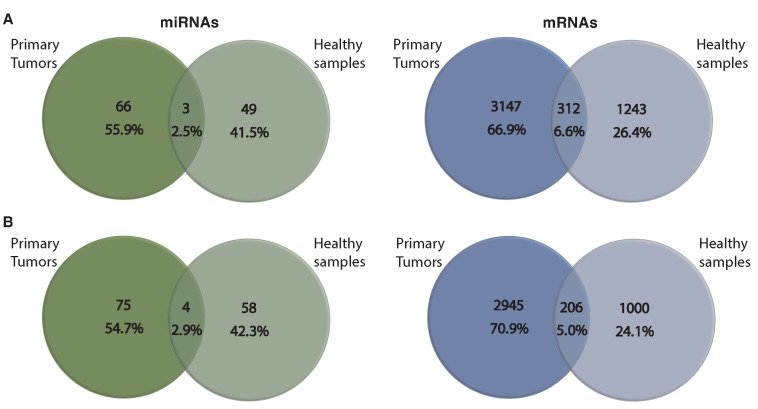
Venn diagram of overlap detected miRNA and mRNA signatures by MSRFR using primary tumors and healthy samples of (**A**) lung and (**B**) kidney.

We also applied MSRFR on healthy samples of kidney and lung that are deposited in TCGA and compared the findings to the two cohort-specific and their shared tissue-specific first regulatory modules. We found marginal overlaps between healthy and disease samples (Figure 6), indicating that MSRFR effectively determines miRNA–mRNA signatures related to primary tumors.

### 3.5. Literature Validation of Identified miRNA–mRNA Signatures

**Figure 7:**
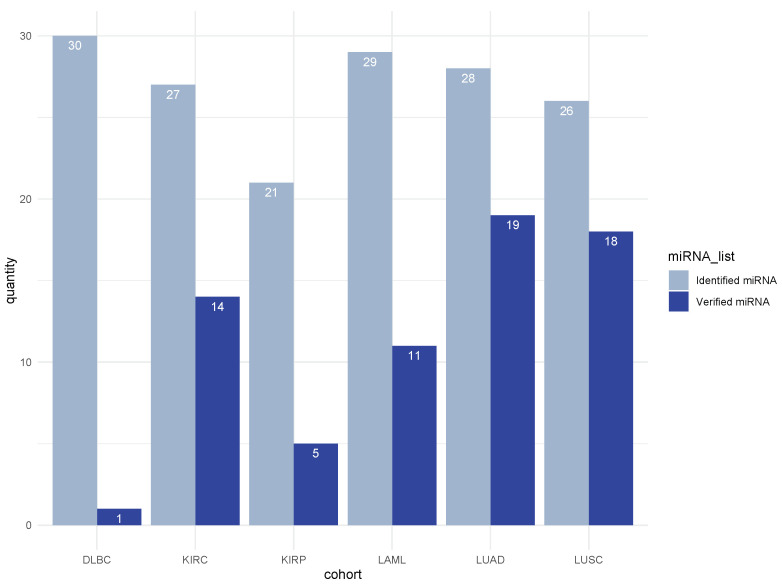
Selected miRNAs and those with literature validation support.

We investigated the relevance of the identified cohort-specific regulatory modules underlying cancer using a literature survey validation by checking whether identified miRNAs are important for formation or progression of the corresponding cancer type. For 68 selected miRNAs out of 161 identified miRNAs (42.24%) in the first regulatory module of six different cohorts, we found published records in PubMed. The ratio of selected miRNAs with literature support in LUAD and LUSC cohorts was higher than in the other cohorts (Figure 7). Detailed information on all 68 miRNAs is reported in Supplementary Table S2, indicating that MSRFR effectively determines cancer-related miRNAs in cohort-specific regulatory modules.

Moreover, to see if identified miRNA–mRNA signatures are known in the literature (PubMed), we investigated all possible duplex combinations of the top 2 and top 10 selected miRNAs and mRNAs of each cohort/tissue. Specifically, we looked for direct or indirect associations as biomarkers in the formation, progression, or treatment of cancer. For example, mir-152 and mir-30e, the top two selected miRNAs by MSRFR for LUSC cohort, have been shown to improve the non-invasive diagnosis of renal cell carcinoma [35]. mir-142 and APAF1 as target gene are proposed as a promising non-invasive diagnostic biomarker of hepatocellular carcinoma [36], which have been detected by MSRFR for kidney tissue. ASXL2 and BPTF were suggested for a potential therapeutic approach for human diseases [37], and MSRFR identified both in lung tissue. In total, we were able to validate 30 records in PubMed, including miRNA–miRNA, miRNA–mRNA, and mRNA–mRNA interactions, which are listed in Supplementary Table S3.

### 3.6. Abundance of Transcription Factor mRNAs in miRNA–mRNA Signatures

To check if TF expression could be affected in the investigated primary tumors and their specific miRNA–mRNA signatures, we assessed the currently known 1639 human TFs [32] in our nine regulatory modules (first regulatory module in each cohort/tissue). MSRFR found 1030 mRNAs on average in these nine regulatory modules (Supplementary Table S1).

**Figure 8:**
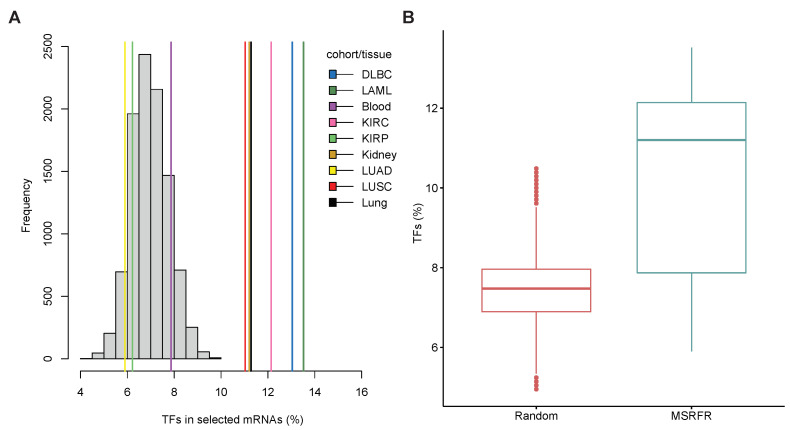
(**A**) Histogram of TFs that are present in each investigated primary tumors and their shared tissue-specific overlaps (blood, kidney, lung). Grey bars represent randomized results of 10,000 gene lists, and colored lines show average TFs in key regulatory modules identified by MSRFR. (**B**) Box plot of the average TFs detected by MSRFR vs. identified in random gene lists.

To test if the number of overlapping TFs is meaningful, we developed a permutation test and compared our result with random sets of genes. To this end, we randomly picked 1030 genes 10,000× and compared the percentage of TFs among random experiments and MSRFR results. The maximum average of randomly identified TFs was 10.49%. In 6 out of 9 cohorts/tissues the average number of TFs identified by MSRFR deterministically dominates the average number of TFs in random genes (Figure 8A). We also found that the difference in the median of TFs in random genes and selected mRNAs by MSRFR is significantly greater (Mann-Whitney test *p*-value = 0.004) than in random sets (Figure 8B).

The detailed information on detected TFs in regulatory modules are listed in Supplementary Table S4.

## 4. Discussion

Understanding the underlying biological mechanisms of primary tumors is crucial for predicting how tumors respond to therapies. miRNAs’ interactions with mRNAs have a major effect on many biological processes that are important in the formation and progression of cancer. Therefore, identifying both cohort- and tissue-specific miRNA–mRNA regulatory modules of cancers have received considerable interest due to its importance in cancer biology.

This study introduces a pipeline to extract tissue- and cohort-specific miRNA–mRNA regulatory modules of multiple cancer types from the same origin using matched miRNA and mRNA expression profiles of primary tumors. We generated a multitask sparse regularized factor regression model which was able to successfully extract and distinguish tissue- and cohort-specific regulatory modules and estimate the effective numbers of cohort-specific and tissue-specific regulatory modules.

Out of all six considered cohorts, MSRFR model outperformed single-task regression method in 5/6 cohorts (see Table 2). We were able to identify mRNAs that are related to tissue type and that were enriched in disease-relevant terms. The identified miRNAs were also reported in the investigated primary tumors, and finally, TFs in the determined miRNA–mRNA signatures indicate their strong involvement in pathogenesis. The sets of experiments with cohorts from unrelated tissues to the phenotype showed marginal overlaps. This indicates that MSRFR determines significant differences between cohorts and identifies tissue-specific modules from the same tissue.

Collectively, these results show that the proposed model is highly effective in identifying key miRNA–mRNA regulatory modules and distinguishing cohort-specific and tissue-specific regulatory modules.

There is an abundant room for further progress in determining similarities in molecular patho-mechanisms. Extensions of this study can be applied to investigate other diseases where similar primary cells are involved to find cohort-specific mechanisms together with the mechanisms shared among underlying conditions. Moreover, further work is required to establish complementary research on other RNAs in the non-coding genome, such as long non-coding RNAs, to decode their functional similarities in different conditions.

## Figures and Tables

**Table 1:** Description of symbols used in the proposed MSRFR model.

Symbol	Definition
Y	mRNA expression profile matrix
X	miRNA expression profile matrix
W	Weight matrix of regression model
E	Error term matrix
$W_{X}$	Weight matrix to project miRNA profiles into low-dimensional space
$W_{Y}$	Weight matrix of linear regression in projected space
*N*	Number of tumors
*D*	Number of miRNAs
*T*	Number of mRNAs
*R*	Dimensionality of projected space
*k*	Index of cohorts (1,...,K)
*S*	Index of tissue (shared between cohorts)
${∥\cdot ∥}_{F}$	Frobenius norm
${∥\cdot ∥}_{1,1}$	$\ell_{1,1}$ norm
$\lambda_{1},\cdots ,\lambda_{4}$	Regularization parameters

**Table 2:** Predictive performance values of MSRFR vs. single-task algorithm on six data sets incorporated in this study, in terms of average NRMSE over mRNAs and their selected ranks. Improved performance is highlighted with bold fonts.

	MSRFR		Single-Task	
**Cohort**	**Rank**	**NRMSE**	**Rank**	**NRMSE**
DLBC	13	0.7664	19	**0.5803**
LAML	19	**0.6708**	20	0.7479
KIRC	18	**0.7412**	20	0.9796
KIRP	16	**0.7626**	20	0.7838
LUAD	20	**0.7840**	20	0.8832
LUSC	19	**0.8039**	20	0.8870

**Table 3:** Number of identified regulatory modules and regulatory modules with significant survival differences, found by MSRFR and single-task algorithm for cohorts incorporated in this study.

	MSRFR		Single-Task	
**Cohort or Tissue**	**All**	**Survival**	**All**	**Survival**
DLBC	3	1	19	0
LAML	9	3	20	4
Blood	10	3	-	-
KIRC	8	6	20	8
KIRP	6	2	20	0
Kidney	10	6	-	-
LUAD	10	4	20	14
LUSC	9	1	20	2
Lung	10	1	-	-
Total (%)	75	27 (36)	119	28 (23.53)

## Data Availability

Publicly available data sets were analyzed in this study. These data sets can be found at https://portal.gdc.cancer.gov, accessed on 10 August 2022. Our implementation of proposed MSRFR algorithm in R is available at https://github.com/MiladMokhtaridoost/MSRFR, accessed on 10 August 2022. Gene set enrichment analysis was conducted using publicly available Metascape web tools at https://metascape.org, accessed on 10 August 2022.

## Supplementary Materials

The following are available online at https://www.mdpi.com/article/10.3390/cancers14194939/s1, Supplementary Table S1: Identified miRNAs–mRNA list, Supplementary Table S2: Supporting evidence for cohort-specific identified miRNAs, Supplementary Table S3: Supporting evidence for importance of interaction between identified miRNAs and mRNAs, Supplementary Table S4: Verification of identified TFs.

## Abbreviations

    The following abbreviations are used in this manuscript:

miRNA	MicroRNA
mRNA	Messenger RNA
MSRFR	Multitask Learning Sparse Regularized Factor Regression
NRMSE	Normalized Root Mean Squared Error
TCGA	The Cancer Genome Atlas
DLBC	Lymphoid neoplasm diffuse large B-cell lymphoma
LAML	Acute myeloid leukemia
KIRC	Kidney renal clear cell carcinoma
KIRP	Kidney renal papillary cell carcinoma
LUAD	Lung adenocarcinoma
LUSC	Lung squamous cell carcinoma
TF	Transcription Factor

## Appendix A

$$\begin{array}{cc} h(W_{XS},W_{YS}) & =\frac{\partial }{\partial W_{XS}}\left(\frac{1}{2}\sum_{k=1}^{K}∥Y_{k}-X_{k}W_{XSk}W_{YSk}{∥}_{F}^{2}+\lambda_{2}{∥W_{XS}∥}_{F}^{2}\right)\\ \; & =\sum_{k=1}^{K}\left(X_{k}^{⊤}X_{k}W_{XSk}W_{YSk}W_{YS}^{⊤}-X_{k}^{⊤}Y_{k}W_{YS}^{⊤}\right)+2\lambda_{2}W_{XS}\\ h(W_{Xk},W_{Yk}) & =\frac{\partial }{\partial W_{Xk}}\left(\frac{1}{2}∥Y_{k}-X_{k}W_{XSk}W_{YSk}{∥}_{F}^{2}+\lambda_{2}{∥W_{Xk}∥}_{F}^{2}\right)\\ \; & =-X_{k}^{⊤}Y_{k}W_{Yk}^{⊤}+X_{k}^{⊤}X_{k}W_{XSk}W_{YSk}W_{Yk}^{⊤}+2\lambda_{2}W_{Xk}\\ g(W_{XS},W_{YS}) & =\frac{\partial }{\partial W_{YS}}\left(\frac{1}{2}\sum_{k=1}^{K}∥Y_{k}-X_{k}W_{XSk}W_{YSk}{∥}_{F}^{2}+\lambda_{4}{∥W_{YS}∥}_{F}^{2}\right)\\ \; & =\sum_{k=1}^{K}\left(W_{XS}^{⊤}X_{k}^{⊤}X_{k}W_{XSk}W_{YSk}-W_{XS}^{⊤}X_{k}^{⊤}Y_{k}\right)+2\lambda_{4}W_{YS}\\ g(W_{Xk},W_{Yk}) & =\frac{\partial }{\partial W_{Yk}}\left(\frac{1}{2}∥Y_{k}-X_{k}W_{XSk}W_{YSk}{∥}_{F}^{2}+\lambda_{4}{∥W_{Yk}∥}_{F}^{2}\right)\\ \; & =-W_{Xk}^{⊤}X_{k}^{⊤}Y_{k}+W_{Xk}^{⊤}X_{k}^{⊤}X_{k}W_{XSk}W_{YSk}+2\lambda_{4}W_{Yk} \end{array}$$

## Appendix B

(A1)
$$\begin{array}{cc} \; & ∥h\left(W_{XS},W_{YS}^{\left(t+1\right)}\right)-h\left(W_{XS}^{\prime },W_{YS}^{\left(t+1\right)}\right){∥}_{F}\\ \; & =\sum_{k=1}^{K}\left(X_{k}^{T}X_{k}\left[\left(W_{XS}-W_{XS}^{\prime }\right)\;W_{Xk}\right]W_{YSk}^{\left(t+1\right)}W_{YS}^{T(t+1)}\right)+2\lambda_{2}\left(W_{XS}-W_{XS}^{\prime }\right)\\ \; & \leq (\sum_{k=1}^{K}∥X_{k}^{T}X_{k}W_{Xk}{∥}_{F}∥W_{YSk}^{\left(t+1\right)}W_{YS}^{T(t+1)}{∥}_{F}+2\lambda_{2})∥W_{XS}-W_{XS}^{\prime }{∥}_{F} \end{array}$$

(A2)
$$\begin{array}{cc} \; & ∥h\left(W_{Xk},W_{Yk}^{\left(t+1\right)}\right)-h\left(W_{Xk}^{\prime },W_{Yk}^{\left(t+1\right)}\right){∥}_{F}\\ \; & =X_{k}^{T}X_{k}\left[W_{XS}\;\left(W_{Xk}-W_{Xk}^{\prime }\right)\right]W_{YSk}^{\left(t+1\right)}W_{Yk}^{T(t+1)}+2\lambda_{2}\left(W_{Xk}-W_{Xk}^{\prime }\right)\\ \; & \leq (∥X_{k}^{T}X_{k}W_{XS}{∥}_{F}∥W_{YSk}^{\left(t+1\right)}W_{Yk}^{T(t+1)}{∥}_{F}+2\lambda_{2})∥W_{Xk}-W_{Xk}^{\prime }{∥}_{F} \end{array}$$

(A3)
$$\begin{array}{cc} \; & ∥g\left(W_{XS}^{\left(t\right)},W_{YS}\right)-g\left(W_{XS}^{\left(t\right)},W_{YS}^{\prime }\right){∥}_{F}\\ \; & =∥\sum_{k=1}^{K}\left(W_{XS}^{T(t)}X_{k}^{T}X_{k}W_{XSk}^{\left(t\right)}{\left[{\left(W_{YS}-W_{YS}^{\prime }\right)}^{T}\;W_{Yk}^{T}\right]}^{T}\right)+2\lambda_{4}\left(W_{YS}-W_{YS}^{\prime }\right){∥}_{F}\\ \; & \leq (\sum_{k=1}^{K}∥W_{XS}^{T(t)}X_{k}^{T}X_{k}{\left[W_{XS}\;W_{Xk}W_{Yk}\right]}^{\left(t\right)}{∥}_{F}+2\lambda_{4})∥W_{YS}-W_{YS}^{\prime }{∥}_{F} \end{array}$$

(A4)
$$\begin{array}{cc} \; & ∥g\left(W_{Xk}^{\left(t\right)},W_{Yk}\right)-g\left(W_{Xk}^{\left(t\right)},W_{Yk}^{\prime }\right){∥}_{F}\\ \; & =∥W_{Xk}^{T(t)}X_{k}^{T}X_{k}W_{XSk}^{\left(t\right)}{\left[W_{YS}^{T}\;{\left(W_{Yk}-W_{Yk}^{\prime }\right)}^{T}\right]}^{T}+2\lambda_{4}\left(W_{Yk}-W_{Yk}^{\prime }\right){∥}_{F}\\ \; & \leq (∥W_{Xk}^{T(t)}X_{k}^{T}X_{k}{\left[W_{XS}W_{YS}\;W_{Xk}\right]}^{\left(t\right)}{∥}_{F}+2\lambda_{4})∥W_{Yk}-W_{Yk}^{\prime }{∥}_{F} \end{array}$$

## Appendix C

**Algorithm A1** Optimization algorithm
Input: ${\left\{X_{k}\right\}}_{k=1}^{K}\in R^{N_{k}\times D}$, ${\left\{Y_{k}\right\}}_{k=1}^{K}\in R^{N_{k}\times T}$, $\lambda_{1}\in R_{+}$, $\lambda_{2}\in R_{+}$, $\lambda_{3}\in R_{+}$, $\lambda_{4}\in R_{+}$, $R_{U}\in Z_{++}$ Output: $W_{XS}^{☆}$, $W_{YS}^{☆}$, $R_{S}^{☆}$, ${\left\{W_{Xk}^{☆},W_{Yk}^{☆},R_{k}^{☆}\right\}}_{k=1}^{K}$ $R_{S},{\left\{R_{k}\right\}}_{k=1}^{K}\leftarrow R_{U}$ ρ=0 **while** ρ==0 **do** t←0 ${\left\{W_{Xk}\right\}}_{k=1}^{K}\leftarrow$ a random matrix from $R^{D\times R_{k}}$ $W_{XS}\leftarrow$ a random matrix from $R^{D\times R_{S}}$ **while** optimization problem (2) not converged **do** $W_{XS}^{\left(t+1\right)}\leftarrow$ update $W_{XS}$ using (3a) ${\left\{W_{Xk}^{\left(t+1\right)}\right\}}_{k=1}^{K}\leftarrow$ update ${\left\{W_{Xk}\right\}}_{k=1}^{K}$ using (3b) $W_{YS}^{\left(t+1\right)}\leftarrow$ update $W_{YS}$ using (3c) ${\left\{W_{Yk}^{\left(t+1\right)}\right\}}_{k=1}^{K}\leftarrow$ update ${\left\{W_{Yk}\right\}}_{k=1}^{K}$ using (3d) t←t+1 **end while** **if** $\mathrm{rank}\left(W_{XS}^{\left(t+1\right)}\right)<R_{S}$ **or** $\mathrm{rank}\left(W_{YS}^{\left(t+1\right)}\right)<R_{S}$ **then** $R_{S}\leftarrow R_{S}-1$ **else** ρ++ **end if** **for** k=1 to *K* **do** **if** $\mathrm{rank}\left(W_{Xk}^{\left(t+1\right)}\right)<R_{k}$ **or** $\mathrm{rank}\left(W_{Yk}^{\left(t+1\right)}\right)<R_{k}$ **then** $R_{k}\leftarrow R_{k}-1$ **else** ρ++ **end if** **end for** $\rho =\left\lfloor \frac{\rho }{K+1}\right\rfloor$ **end while** $W_{XS}^{☆}\leftarrow W_{XS}^{\left(t+1\right)}$, ${\left\{W_{Xk}^{☆}\right\}}_{k=1}^{K}\leftarrow {\left\{W_{Xk}\right\}}_{k=1}^{K}$ $W_{YS}^{☆}\leftarrow W_{YS}^{\left(t+1\right)}$, ${\left\{W_{Yk}^{☆}\right\}}_{k=1}^{K}\leftarrow {\left\{W_{Yk}\right\}}_{k=1}^{K}$ $R_{S}^{☆}\leftarrow R_{S}$, ${\left\{R_{k}^{☆}\right\}}_{k=1}^{K}\leftarrow {\left\{R_{k}\right\}}_{k=1}^{K}$
